# Pilot randomised controlled trial of the ENGAGER collaborative care intervention for prisoners with common mental health problems, near to and after release

**DOI:** 10.1186/s40814-017-0163-6

**Published:** 2017-07-07

**Authors:** Charlotte Lennox, Tim Kirkpatrick, Rod S. Taylor, Roxanne Todd, Clare Greenwood, Mark Haddad, Caroline Stevenson, Amy Stewart, Deborah Shenton, Lauren Carroll, Sarah L. Brand, Cath Quinn, Rob Anderson, Mike Maguire, Tirril Harris, Jennifer Shaw, Richard Byng

**Affiliations:** 1 Division of Psychology and Mental Health, The University of Manchester, 2.315 Jean McFarlane Building, Oxford Road, Manchester, M13 9PL UK; 2 Plymouth University Peninsula Schools of Medicine and Dentistry, N9 Plymouth Science Park, Davy Road, Plymouth Science Park, Derriford, Plymouth, PL6 8BX UK; 3 Division of Psychology and Mental Health, The University of Manchester, 2.309 Jean McFarlane Building, Oxford Road, Manchester, M13 9PL UK; 4 The Medical School, The University of Sheffield, Beech Hill Road, Sheffield, S10 2RX UK; 5 Centre for Mental Health Research, School of Health Sciences, City, University of London, Northampton Square, London, EC1V 0HB UK; 6 Institute of Health Research, University of Exeter Medical School, University of Exeter, South Cloisters, St Luke’s campus, Heavitree Road, Exeter, EX1 2LU UK; 7 Centre for Criminology, University of South Wales, Pontypridd, CF37 1DL UK; 8 King’s College London, Strand, London, WC2R 2LS UK; 9 Division of Psychology and Mental Health, The University of Manchester, 2.317 Jean McFarlane Building, Oxford Road, Manchester, M13 9PL UK

**Keywords:** Prison, Offender, Mental health, Pilot, Randomised controlled trial

## Abstract

**Background:**

Rates of common mental health problems are much higher in prison populations, but access to primary care mental health support falls short of community equivalence. Discontinuity of care on release is the norm and is further complicated by substance use and a range of social problems, e.g. homelessness. To address these problems, we worked with criminal justice, third sector social inclusion services, health services and people with lived experiences (peer researchers), to develop a complex collaborative care intervention aimed at supporting men with common mental health problems near to and following release from prison. This paper describes an external pilot trial to test the feasibility of a full randomised controlled trial.

**Methods:**

Eligible individuals with 4 to 16 weeks left to serve were screened to assess for common mental health problems. Participants were then randomised at a ratio of 2:1 allocation to ENGAGER plus standard care (intervention) or standard care alone (treatment as usual). Participants were followed up at 1 and 3 months’ post release. Success criteria for this pilot trial were to meet the recruitment target sample size of 60 participants, to follow up at least 50% of participants at 3 months’ post release from prison, and to deliver the ENGAGER intervention. Estimates of recruitment and retention rates and 95% confidence intervals (CIs) are reported. Descriptive analyses included summaries (percentages or means) for participant demographics, and baseline characteristics are reported.

**Results:**

Recruitment target was met with 60 participants randomised in 9 months. The average retention rates were 73% at 1 month [95% CI 61 to 83] and 47% at 3 months follow-up [95% CI 35 to 59]. Ninety percent of participants allocated to the intervention successfully engaged with a practitioner before release and 70% engaged following release.

**Conclusions:**

This pilot confirms the feasibility of conducting a randomised trial for prison leavers with common mental health problems. Based on this pilot study and some minor changes to the trial design and intervention, a full two-centre randomised trial assessing the clinical and cost-effectiveness of the ENGAGER intervention is currently underway.

**Electronic supplementary material:**

The online version of this article (doi:10.1186/s40814-017-0163-6) contains supplementary material, which is available to authorized users.

## Background

Offenders, especially prisoners, have a high prevalence of common mental health problems (e.g. depression, anxiety). Rates of 50–90% for all mental health problems have been found in UK [1, 2] and international [3] prison populations, with high levels of unemployment, relationship problems and past trauma [4, 5]. Prison has been identified as an opportunity for treatment but release into chaotic community environments poses a challenge for treatment and also research [6]. Critical Time Intervention has been developed and evaluated for prisoners with severe mental illness and shown to have a significant impact at 6 weeks and 6 months post release from prison in terms of increasing engagement with services [7]. No studies have attempted to evaluate such ‘through the gate’ mental health interventions for prisoners with common mental health problems.

In 1996 ‘*Patient or Prisoner*’, a Discussion Paper by Her Majesty’s Inspectorate of Prisons, highlighted the inadequacies in prison healthcare in England and Wales and argued for equivalence, stating explicitly that prisoners should be entitled to the same level of healthcare as that provided to the general public in the community [8]. Prison mental health in-reach teams were established in England and Wales over the last decade for prisoners with severe and enduring mental illness. However, mental health in-reach services fall short of community equivalence, with wide variation in service provision and that services for common mental health problems in prison are even more limited [9–11]. Although the UK government developed the Improving Access to Psychological Therapies (IAPT) for depression and anxiety, these services have been poorly resourced in prisons, with pharmacological interventions often being the only treatment provided [12]. This is despite some evidence that psychological interventions, even ‘low intensity’ treatments, may be as effective for offenders as they are for the general population [13]. An observational, prospective cohort study evaluating IAPT for prisoners found clinical recovery being achieved in 55% of depression and 52% of anxiety cases [12]. However, while this is similar to results in community IAPT demonstration sites in Newham and Doncaster [14] effect sizes in observational studies do not account for regression to the mean.

Discontinuity of care on release from prison is the norm [4]. Once released, ex-prisoners with common mental health problems are, in theory, provided for by mainstream statutory services including general practice and IAPT services. In reality few access these services [6]. Many prisoners with common mental health problems have substantial co-morbidity with personality disorders and substance misuse [2, 6] and these offenders may fall between the cracks in service provision between general practice, IAPT and substance misuse services [15–18]. Offenders are further disadvantaged by their resistance to seeking help and to accepting mental health diagnoses, with lower levels of GP registration and high rates of personal and social problems such as homelessness and relationship difficulties [4–6, 19–22].

To address these multiple problems, we have developed a complex collaborative care intervention aimed at supporting male prisoners with common mental health problems near to and following release (ENGAGER), working with criminal justice providers (prison and community), third sector social inclusion services, health services and people with lived experiences (peer researchers), using a range of methodologies [23]. The underpinning principles and practices of ENGAGER are (a) to develop trust and engagement through showing respect and giving practical support; (b) to support mental health through a psychological therapy informed ‘shared understanding and action plan’ and mentalisation-based approaches which are not disorder specific or based on the ability to turn up for weekly appointments; and (c) to supporting individuals to achieve their personal goals through alignment of personal strengths, family and community resources and joint work with criminal justice, third sector and other health providers.

Mentalising is a natural human ability. It is the capacity to think about our own mind and the minds of others and understand how emotions, thoughts, wishes and impulses lie behind and influence our behaviour and the behaviour of others. Good mentalising involves being able to acknowledge that often we do not accurately know what people are thinking and feeling but that often we can make more or less accurate guesses. Good mentalising also involves having an authentic interest in other peoples’ emotions and thoughts and not making quick assumptions about why a person may have behaved as they did. For example, rather than assuming why your client has started to drink again, instead being open and curious to exploring with them what was happening for them.

Prisons are complex and difficult environments in which to conduct research, and simply getting access can be difficult for researchers. Rigorous evaluation designs such as randomised controlled trials (RCT) can be challenging to implement without sufficient piloting. In addition, studies following recently released prisoners can be affected by low retention rates [24]. Therefore, for all these reasons, it is essential that trial methods are tested prior to a definitive trial.

This pilot trial aimed to investigate whether it was possible to recruit and retain prisoners with common mental health problems in a RCT of the ENGAGER intervention. The specific objectives of the pilot trial were to address feasibility issues and uncertainties about undertaking a definitive RCT of the ENGAGER intervention: inclusion and exclusion criteria, adequate rates of eligibility, consent and randomisation, acceptable rates of participation in the intervention, ability to maintain researcher blinding to trial arm allocation, ability to retain participants at 1 and 3 months post release from prison; and to provide sufficient levels of completion of outcome measures. Success criteria for this pilot trial were to recruit 60 people to the study (sample size sufficient to test trial procedures), to deliver the ENGAGER intervention, and to follow up at least 50% of all participants 3 months after release.

In line with the Medical Research Council framework [25] for the development and evaluation of complex interventions, this paper describes the process of the pilot trial and lessons for trial science.

## Methods

This external pilot trial is reported in accord with the CONSORT 2010 statement: extension to randomised pilot and feasibility trials (Additional file 1).

### Design

This pilot study undertook a parallel two-group RCT design with participants allocated to either the ENGAGER intervention or treatment as usual (TAU) with a parallel mixed methods process evaluation.

### Setting

The study took place in two prisons housing adult male prisoners only in two regions (North West and South West) of England.

### Participants

Prison records were searched to identify an initial list of potential participants. Those eligible had to be male, currently serving a prison sentence of up to and including 2 years, within four to 16 weeks of their release date, and planning to resettle within the geographical area of the study. Participants were ineligible if they were identified by reviewing clinical records and in discussion with the prison in-reach team as having a severe and enduring mental illness, and/or were on the caseload of the prison mental health in-reach team, and/or were on the caseload of the Offender Personality Disorder Pathway programme. Those who presented a serious risk of harm to the researchers or intervention practitioners, and those unable to provide informed consent, were also excluded.

The rational for only including males was both pragmatic and clinical. The number of females in prison is much smaller, and the prisons are geographically remote, therefore impacting the feasibility of a trial.

At the North West site, eligible participants were approached by a Clinical Studies Officer from the Clinical Research Network, and at the South West site, they were approached by one of the researchers. In the South West, the research team had gained approval to approach participants directly, arguing it is inappropriate to use prison staff, including prison healthcare staff, to make the first approach because of the potentially coercive (or perceived coercive) nature of the relationship in the prison environment.

Potential participants were approached verbally and then, if willing to consider participation, provided with written information about the study and an opportunity for further discussion. Having had the opportunity to discuss their involvement in the study and ask questions, potential participants were asked to sign the consent form if they were willing to take part.

### Screening for recruitment into the trial

The screening interview was delivered in a narrative conversational format to support rapport building. It incorporated the following standardised screening measures which were read aloud to all participants in order to avoid any potential embarrassment regarding reading difficulties:

#### Patient health questionnaire (PHQ-9) [26]

The PHQ-9 is a nine-item scale for depressive disorder. It asks about feelings of anhedonia, low mood, sleep disturbances, poor appetite, low self-esteem, psychomotor retardation and suicidal ideation in the last 2 weeks. Participants were screened in if they reached a score of 10 or more.

#### Generalised Anxiety Disorder (GAD-7) [27]

The GAD-7 is a seven-item screening tool and severity measure of generalised anxiety disorder; it covers feelings of fear, worry, restlessness and irritability in the last 2 weeks. Participants were screened in if they reached a score of 10 or more.

#### Primary care post-traumatic stress screening scale (PTSD) [28]

The PTSD screens whether a person is presenting with PTSD symptoms as a result of a traumatic experience. The scale is based on four main symptoms of PTSD of which three or more had to be present to screen in.

#### Past/future common mental health problem identification

This screen was developed to capture individuals who may appear well in prison but have struggled with common mental health issues before prison and/or are likely to again after release [29]. It reports whether a person has experienced a common mental health problem including depression, anxiety and post-traumatic stress during the past 2 years which prevented them from functioning normally in everyday tasks, *or* if they thought this was likely to be a problem for them following release. This screen was adapted further during the pilot to be more stringent as it became evident that many people reported experiencing symptoms in the past only. Therefore, as the pilot progressed, participants were screened in on this only if they had experienced problems compatible with a common mental health problem during the past 2 years, which prevented them from functioning normally in everyday tasks *and* if they thought this was going to be a problem for them following release.

Participants had to screen in on at least one of the four instruments to be included in the study. Participants who screened in were given an additional information sheet, which explained the RCT. The researcher ensured that the potential participant fully understood the randomisation process, and reiterated that participation was voluntary, that they could withdraw at any time and the arrangements to ensure confidently and data protection. Based on our group work with peer researchers, they recommended that we explain the randomisation process to participants as being undertaken by a computer programme, rather than use comparisons with ‘flipping a coin’ in which human involvement suggests the potential for tampering. Having had the opportunity to discuss their involvement in the study, participants were asked to sign a second consent form if they were willing to take part.

The screening interview lasted 15–20 min, and at the end, people were informed if they had screened in or not. People screening in continued straight into the baseline interview and for those screening out this was the end of their participation in the study.

### Baseline interview

The baseline interview lasted about 40 min and consisted of a range of different questionnaires and semi-structured interviews administered in order to test them for acceptability for inclusion in the full trial. The testing of these measures and decisions for inclusion in the trial is not reported in this study, but will be reported elsewhere. The baseline interview was administered by the same researcher and delivered in a conversational style. Demographic information was also collected and included information from the following domains: age, ethnicity, education, employment, housing and benefits. Current offence, sentence length and offence history were also collected.

### Randomisation process

Participants identified with either current common mental health problems or probable common mental health problems upon release were randomised at a ratio of 2:1 allocation to either ENGAGER plus standard care (intervention group) or standard care alone (TAU group). To ensure concealment, randomisation was carried out by means of a web-based system developed and maintained by Peninsula Clinical Trials Unit. Communication of randomisation went to the lead researcher at each site, by automatic email. The researcher who performed the screening and baseline interview was blind to the participant’s group allocation; a second researcher visited the participant in prison to deliver a letter informing him to which group he been randomised.

#### Intervention

The ENGAGER intervention sets up a pathway of care up to 12 weeks prior to their release and for 3 to 5 months in the community. The intervention aims to overcome a set of challenges that have been identified as being problematic in this group including:

- Barriers associated with the transition when leaving prison and re-entering the community
- The provision of services designed to meet a single diagnostic need (e.g. depression) or social problem (e.g. homelessness) rather than the reality of people with multiple and complex needs
- Participants’ reluctance to trust services (or to see themselves as having mental health problems)

During the pilot trial, ENGAGER practitioners and supervisors met these challenges by:

- Working on individuals’ strengths to develop a shared understanding and shared plan addressing their complex needs
- Adopting a pragmatic therapeutic approach, incorporating a mentalisation-based approach alongside existing skills
- Release day working, such that each person is met at the gate and taken to their service appointments, accommodation etc. on that day
- Flexible one-to-one working including use of text messaging, practical support, crisis support and planned therapeutic work
- Using the full range of resource components—individual strengths, family and community resources, practitioner skills and additional resources/agencies in prison and the community to meet individualised goals
- Actively liaising with other services such as substance misuse teams, general practitioners and services relating to housing, employment and benefits

The Engager supervisor and practitioner meet jointly with the individual on at least two occasions in prison and once in the community to engage with individuals and to develop and review the shared understanding. Engager practitioners meet with individuals at least weekly in prison and the community after release for 8–16 weeks, according to their needs. Practitioners actively review progress, assertively follow up and liaise with others involved in the individual’s care and resettlement including families, peer mentors and other agencies and organisations identified in the development of the shared plan. Practitioners plan, work towards and deliver a positive ending. In contrast, treatment as usual consists of general practice contact for some and rarely psychological therapy. Those with opiate addiction are often seen frequently by substance misuse services.

#### Treatment as usual group

Individuals in the TAU group were able to access primary care, mental health and substance misuse services in the standard way while in prison. They also received support from criminal justice and any other third sector organisations in the standard way in the community.

### Follow-up

After the baseline assessment meeting, the same researcher had contact with the participant on at least three further occasions. Throughout this process, attempts were made to maintain blinding to trial arm allocation, and a log was made of occasions when the researcher became aware of it. About a week before the participant’s release, a meeting was held; the main objective of which was to strengthen the researcher-participant relationship and thereby enhance follow-up rates. During this meeting, the researcher confirmed contact information that had been provided during the baseline interview and made any amendments, e.g. where phone number, addresses and contact with services had changed.

Participants were then followed up at 1 and 3 months post release by the same researcher. At approximately 1 month post release, the researcher contacted and spoke to the participant either via a phone call or (preferably) face-to-face. The main objective was again to sustain engagement and plan further contact. At this meeting the researcher discussed the 3-month follow-up in detail and agreed the best way to contact the participant for that appointment. The researcher also obtained any new mobile phone numbers if contact had been made without an up to date mobile contact, or any new addresses or services the person may now be in contact with.

The 3-month follow-up took place between 8 and 15 weeks post release, although researchers endeavoured to complete data collection as close to the 3-month point as possible. Researchers normally contacted the participants by phone or via a service they were in contact with, e.g. probation and arranged to meet them at a convenient location in the community. Where possible, interviews were conducted in the premises of services with which the participant was engaging in order to make this as convenient as possible. Where this was not possible, researchers arranged to conduct the interviews in a suitable location in the community and adhered to the Lone Working policy, being accompanied by a ‘buddy’ as an additional safeguard, if required.

As with the baseline data collection, the researcher continued to deliver the follow-up data collection interview using narrative conversational format. The same assessments at baseline were repeated at this follow-up. However, not all participants completed the same outcome measures due to testing a range of outcomes for acceptability for inclusion in the full trial. The results of the testing of these outcome measures and decisions for inclusion in the full trial will be reported elsewhere and no outcome data is reported in this study.

All researchers received training in Good Clinical Practice and in the requirements of the study protocol. Joint training for researchers at both sites was undertaken prior to commencing recruitment to the trial to ensure a consistency of approach, from consent to data collection. In addition, weekly team meetings via video conference allowed both research sites to ensure a similar quality.

### Statistical analysis

Given the primary feasibility and acceptability objectives of this pilot trial, no within- or between-group inferential comparisons of outcomes were performed. Estimates of recruitment and retention rates and 95% confidence intervals (CIs) are reported. Descriptive analyses included summaries (percentages or means and 95% CIs) for participant demographics and baseline characteristics and each outcome baseline and each follow-up.

## Results

### Recruitment to the trial

**Fig. 1:**
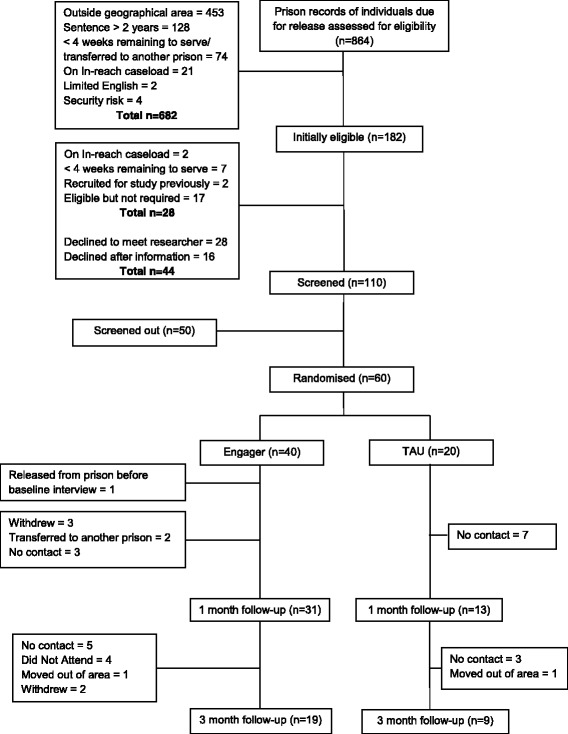
Consort of participant flow

Recruitment commenced in August 2014 and ended in April 2015. Figure 1 shows the participant flow through the study. The records of 864 individuals due for release between September 2014 and April 2015 were examined using prison databases and 21% (*n =* 182) were identified as eligible, according to sentence length, release date and area, and other exclusion criteria, e.g. risk and severe mental illness. Of these 182 initially eligible individuals, a further 28 were excluded prior to assessment for common mental health problems (two were subsequently accepted onto the in-reach team caseload; seven moved into the less than 4 weeks remaining to serve window; two had previously been recruited for the study; and 17 were no longer required as our target of 60 participants for randomisation had been reached). Of the remaining 154, 28 declined to meet the researcher (North West site only following initial approach by Clinical Studies Officer) and 16 declined to consent after meeting one of the researchers (across both sites). Thus 110 people (71%) consented to take part, all of whom completed the screening interview. Of these, 50 (45%) were screened out as not having current common mental health problems or probable common mental health problems upon release.

### Characteristics of pilot trial population

A total of 60 participants screened in as having current common mental health problems or probable common mental health problems upon release. Table 1 below shows the mean scores on all assessments and the number and percentage of participants screening in on each assessment. A total of 12 (20%) participants screened in on all four assessments, 10 (17%) on three assessments, and 18 (30%) on two assessments. Twenty participants (33%) screened in on just one assessment: none on the GAD-7, one on PTSD, two on the PHQ-9, and 17 on the historical screen.

**Table 1:** Descriptives of the screening assessments

	Mean (SD)	*N* (%) screening in
PHQ-9	10.3 (6.02)	32 (53)
GAD-7	8.7 (4.99)	24 (40)
PTSD	2.3 (1.45)	23 (38)
Historical screen		55 (92)

### Baseline

Baseline questionnaires were completed in full for all participants except for one. For this participant it was not possible to complete the screening and baseline interviews on the same day and then was released unexpectedly early.

Table 2 shows the demographic and baseline characteristics of the pilot sample. The majority (95%) of participants were White with an average age of 33 (range 19–57 years old). Unstable accommodation was common with nearly half (*n =* 28; 47%) of participants having spent the majority of time in the last 3 months before coming into prison in some form of temporary accommodation, sofa surfing or homeless and a quarter (*n =* 15; 25%) having lived in more than one type of accommodation in the 3 months before prison. Just over a third (*n =* 21; 36%) had no qualifications and only 1 in 5 participants (*n =* 12; 20%) were in employment in the 3 months before prison. Over 70% (*n =* 42) had an annual income of less than £7500, with the majority (*n =* 43; 80%) receiving benefits.

**Table 2:** Demographics and baseline characteristics

		TAU (*n =* 20)	Intervention (*n =* 39^a^)
Age; mean (SD)		32.3 (10.46)	33.3 (8.51)
Ethnicity; *n* (%)	White	19 (95)	37 (95)
	Other	1 (5)	2 (5)
Relationship; *n* (%)	Married, civil partnership, cohabiting	2 (10)	6 (15)
	Single, divorced, separated	18 (90)	33 (85)
Accommodation; *n* (%)	Own home/rented accommodation	10 (50)	18 (44)
	Temporary accommodation/homeless	10 (50)	18 (44)
	Supported accommodation	0 (0)	3 (7)
Education; *n* (%)	No qualifications	6 (30)	15 (39)
	CSE/ GCSE/ O level	6 (30)	8 (20)
	Further qualifications	8 (40)	16 (41)
Employment; *n* (%)	Employed	6 (30)	6 (15)
	Unemployed	14 (70)	33 (85)
Benefits; *n* (%)	No benefits	8 (40)	8 (20)
	Claiming benefits	12 (60)	31 (80)
Income^b^; *n* (%)	Less than £7500	14 (70)	28 (72)
	More than £7500	4 (20)	10 (26)
Index offence(s)^c^; *n* (%)	Violent	11 (55)	22 (56)
	Non violent	5 (25)	12 (30)
History of self-harm; *n* (%)	Yes	5 (25)	5 (13)
	No	15 (75)	34 (87)
History of self- report drug or alcohol problem; *n* (%)	Yes	15 (75)	25 (64)
	No	5 (25)	14 (36)
Standardised Assessment of Personality score	≥3	17 (85)	33 (85)
	≥5	8 (40)	18 (46)

^a^One participant was released after randomisation but before baseline data could be collected

^b^Two participants in the TAU group and one participant in the Intervention group chose ‘prefer not to say’

^c^Four participants’ in the TAU group and five participants in the Intervention group were missing

Over half (*n =* 33; 56%) of the participants had at least one violent index offence, where ‘violent’ included assault, affray, grievous bodily harm, robbery and possession of an offensive weapon, but not witness intimidation (unless further details are indicated, violence was involved). Only 10% (*n =* 5) of participants had no previous periods of imprisonment, with the average being six previous periods of imprisonment. Substance misuse was common with 68% (*n =* 40) self-reporting a drug or alcohol problem and 10 participants (17%) reported previous self-harm. The Standard Assessment of Personality (SAPAS) [30] was used to identify the possible presence of personality disorder; overall 85% scored 3 or more and 66% scored 5 or more. A score of 3 or more correctly identifies the presence of DSM–IV personality disorder.

### Randomisation

Randomisation was carried out successfully. No participants dropped out at the point of randomisation process, i.e. as a result of the group to which they were allocated, indicating acceptability of the randomisation process. One participant was randomised after screening in for the study but before baseline assessments were completed (this was not per protocol). This participant was then released unexpectedly before baseline assessment could be completed.

Maintaining blinding was highly problematic. Despite being given guidance to the contrary, participants shared their status with researchers whom they regularly came across in the close prison environment. In the North West site, by the time participants were due for their 1-month follow-up, the researchers were aware of the trial allocation of all participants.

### Follow-up

The overall retention rate was 73% (44/60) at 1 month [95% CI 60.99–82.86] and 47% (28/60) at 3 months [95% CI 34.63–59.11]. This included six people who had no/limited contact with the researchers at 1 month but re-engaged at 3 months. For all participants successfully followed up, all outcome data was collected.

#### Engagement in the intervention

The intervention was delivered to 36 out of the 40 participants (90%) allocated to the intervention. Twenty-eight of the 36 participants met with their practitioners in the community following their release.

In the South West, three participants were released from prison or transferred to other prison establishments before being seen by the practitioners and therefore did not have contact with practitioners while in prison. The other 17 participants received an average of 3.5 contacts each (range 0–7) in prison. Thirteen of these participants met with their practitioners in the community with an average of 7.6 contacts (range 1–17). Five of the six participants who were met at the gate continued to have contact with the practitioner in the community.

In the North West, one participant decided that the intervention was not for him and did not have any contacts with the practitioners. The other 19 received an average of four contacts each (range 1–8) in the prison. Fifteen participants met with their practitioner in the community, having an average of 9.3 contacts (range 1–19). In addition to the abovementioned man who withdrew early on, four participants did not receive any contacts in the community: one of these decided not to continue with the intervention near to release; one was receiving intensive drug rehabilitation and therefore not seen, at the request of the drug rehabilitation service; one stopped responding and returned to a different prison; and one could not be located after a period in hospital.

## Discussion

This pilot trial sought to assess whether it was possible to demonstrate acceptable levels of recruitment and retention amongst prison leavers with or likely to have common mental health problems, in order to inform a future randomised controlled trial. It is important to perform pilot feasibility trials when the logistics of a large-scale trial are unclear [31]. There are a limited number of intervention trials within criminal justice settings, and studies conducted in prisons and requiring community follow-ups after release are both rare and particularly problematic.

The required number of participants (*n =* 60) were recruited and randomised within the 9 month timescale set for the pilot trial. Of the people who were assessed for eligibility, the majority were leaving prison to a destination outside the geographical area of where the intervention was delivered. The main trial is addressing this to some an extent, as both the North West and South West sites have extended the release area. In addition, following the introduction of resettlement prisons, prisoners are increasingly likely to be moved to a prison closer to home within the last 6 months of their sentence [32]. These changes should increase the numbers being released to the local area of each prison, and therefore increase eligibility rates.

Participation of individuals eligible to be approached and screened was high, with 71% consenting. Of those who declined to take part, the majority were initially approached via the Clinical Studies Officer and not directly by a member of the research team. Feedback from participants approached by the Clinical Studies Officer indicated that they did not really understand why they were being approached by someone not linked to the research. Also being approached directly by the research team enabled questions about the study to be more thoroughly discussed and hence they were better informed about the project. Face-to-face initial approach from the research team is the chosen method of recruitment for the main trial, and recruitment has been shown to be improved by face-to-face consultations [33–35].

Of the 110 individuals consented, 55% screened in (60 out of 110) as having or likely to have common mental health problems on release. Twenty-eight percent screened in on the basis of the historic common mental health question but not the validated questionnaires (and one of these participants later declined the intervention), raising questions about the appropriateness of this element of the selection process. We have now made changes to the historical common mental health screen by also assessing if participants believe symptoms of common mental health problems are likely to recur following release from prison.

Retention of 73% at 1 month demonstrated our ability to follow up after release. The 47% at 3 months was more disappointing, and we have developed further protocols to deal with changes to plans after release. We found that a flexible follow-up window of 4–12 weeks allows researchers to contact and re-engage more participants and we are using this method in the main trial. Researchers will continue to take multiple contact details at baseline for people at high risk of homelessness or transiency, sending several reminders by text message or phone call and offering alternative ways of engaging (phone interview rather than face-to-face). Losses to follow-up are likely to be reduced further as researchers will follow up participants out of area and approval has been granted to provide ‘thank-you’ vouchers at community follow-up sessions. In addition, we are building closer relationships with other involved agencies (e.g. National Probation Service, Community Rehabilitation Companies, substance misuse and accommodation services), including developing information sharing agreements. All of these being methods likely to improve follow-up rates and obtaining consent to contact.

Blinding of researchers was a key problem. Participants were keen to share their experiences with the researchers and very often experienced the research and practitioner teams as both representing ‘ENGAGER’. To maintain blinding would have required rejecting participants’ very practical requests to share contact information and their commitment to ‘ENGAGER’. We considered a range of possible solutions to maintain blinding, such as using a paper-based self-complete outcome measure for participants but decided against this strategy due to literacy problems and the likely increase in incomplete data. In the main trial the researchers will know trial arm allocation. This is seen very much as a positive, as it allows for the continued building of rapport between the researcher and participant to facilitate follow-up rates and allows the participant to openly share their experiences. The researchers will deliver the primary outcome measure in a protocolised way to minimise bias. All other measures will be delivered in the more conversational style, which had been developed in order to reduce stress within the research process.

In terms of other pilot trial findings: randomisation was accepted and the data completion of outcomes at baseline and follow-up were excellent. Engagement with the intervention, a crucial indicator of viability, was also good exceeding attendance for therapy in IAPT service evaluations [14]. Limitations of the pilot included the use of an outcome dataset which was still being changed and finalised throughout the duration of the pilot. While complete outcome data was collected from all participants successfully followed up, not all participants completed the same outcome measures. The results of the testing of these outcome measures and decisions for inclusion in the definitive trial will be reported elsewhere. Strengths of this study included the use of peer researchers to refine procedures and testing all the key procedures in both prisons proposed for the main trial.

## Conclusion

In conclusion, the pilot demonstrated the potential to successfully run a definitive trial. Recruitment is feasible but takes time due to many prison leavers going back to other areas; follow-up is feasible and requires organisation, tenacity and flexibility from researchers; randomisation and collection of outcomes are far less problematic than ensuring researchers are blind to allocation; the ENGAGER intervention was feasible to deliver and acceptable as demonstrated by high levels of engagement. The full trial started in January 2016 (ISRCTN: 11707331).

## Additional file

Additional file 1: CONSORT checklist of information to include when reporting a pilot trial. (PDF 95 kb)

## Abbreviations

CBT: Cognitive behavioural therapy
CIs: Confidence intervals
GAD-7: Generalised Anxiety Disorder
IAPT: Improving Access to Psychological Therapies
NOMS: National Offender Management Service
PHQ-9: Patient Health Questionnaire
PTSD: Post-traumatic stress disorder
RCT: Randomised controlled trial
SAPAS: Standard Assessment of Personality–Abbreviated scale
TAU: Treatment as usual
